# Exploration of the two-photon excitation spectrum of fluorescent dyes at wavelengths below the range of the Ti:Sapphire laser

**DOI:** 10.1111/jmi.12255

**Published:** 2015-05-06

**Authors:** J Trägårdh, G Robb, R Amor, WB Amos, J Dempster, G McConnell

**Affiliations:** * Centre for Biophotonics, Strathclyde Institute for Pharmacy and Biomedical Sciences, University of StrathclydeGlasgow, U.K.; † MRC Laboratory of Molecular BiologyCambridge, U.K.

**Keywords:** Fluorescent dyes, microscopy, multiphoton microscopy, 3T3 cells

## Abstract

We have studied the wavelength dependence of the two-photon excitation efficiency for a number of common UV excitable fluorescent dyes; the nuclear stains DAPI, Hoechst and SYTOX Green, chitin- and cellulose-staining dye Calcofluor White and Alexa Fluor 350, in the visible and near-infrared wavelength range (540–800 nm). For several of the dyes, we observe a substantial increase in the fluorescence emission intensity for shorter excitation wavelengths than the 680 nm which is the shortest wavelength usually available for two-photon microscopy. We also find that although the rate of photo-bleaching increases at shorter wavelengths, it is still possible to acquire many images with higher fluorescence intensity. This is particularly useful for applications where the aim is to image the structure, rather than monitoring changes in emission intensity over extended periods of time. We measure the excitation spectrum when the dyes are used to stain biological specimens to get a more accurate representation of the spectrum of the dye in a cell environment as compared to solution-based measurements.

## Introduction

Two-photon microscopy is an important tool for imaging biological specimens, because of its inherent sectioning capability and negligible level of photo-bleaching away from the focal plane. Additionally, the use of longer excitation wavelengths reduces scattering, allowing imaging deeper into tissue as compared to one-photon excitation (Denk *et al*., 1990, 2006; Diaspro *et al*., 2005, 2006). For UV excitable dyes in particular, such as DAPI, and a number of intrinsic chromophores such as NADH (Xu *et al*., 1996; Zipfel *et al*., 2003; Li *et al*., 2009;) and tryptophan (Li *et al*., 2009), using two-photon excitation potentially significantly reduces the risk of photo-damage as compared to using the UV light required for one-photon excitation (Sako *et al*., 1997). There are also a number of ratiometric dyes for measuring ion concentrations in the cell (Bliton *et al*., 1993), that are UV excitable, making two-photon excitation potentially advantageous for cell physiology measurements. The most commonly used laser for two-photon microscopy is the ultrashort pulsed Ti:Sapphire laser, with a wavelength emission range of 680–1080 nm. A number of studies (Xu & Webb, 1996; Xu *et al*., 1996; Zipfel *et al*., 2003; Drobizhev *et al*., 2011) have shown that for many UV excitable fluorophores, the two-photon excitation efficiency increases towards shorter wavelengths and is still rising at the point where the lower limit of wavelengths available from the Ti:Sapphire laser is reached. This suggests that there could be an advantage to using visible wavelengths for two-photon excitation of UV excitable chromophores. There have been a number of efforts to improve the fluorescence signal intensity yield from UV excitable dyes by using two-photon excitation wavelengths shorter than 680 nm, generated by optical parametric oscillators (OPOs) (McConnell *et al*., 2003; Balaji *et al*., 2004; Botchway *et al*., 2008; Li *et al*., 2010, 2011; Norris *et al*., 2012b) (with or without frequency doubling or sum-frequency mixing), nonlinear visible light generation in photonic crystal fibres pumped by a Ti:Sapphire laser (Palero *et al*., 2005; Li *et al*., 2009), or a high-power CW laser (Bianchini & Diaspro, 2012) as well as by simultaneous absorption of two different excitation wavelengths in the sample (Quentmeier *et al*., 2009). Such short-wavelength two-photon imaging has been employed both for UV excitable fluorescent dyes (McConnell *et al*., 2003; Bianchini & Diaspro, 2012; Norris *et al*., 2012b) and intrinsic fluorophores such as tryptophan (Palero *et al*., 2005; Li *et al*., 2009, 2010), serotonin (Balaji *et al*., 2004; Botchway *et al*., 2008), haemoglobin (Li *et al*., 2009) and NADH (Li *et al*., 2009). However, these studies are only comparing imaging with visible wavelengths to imaging using near-infrared wavelengths from a Ti:Sapphire laser at a single visible excitation wavelength. The two-photon absorption spectrum does not necessarily match the one-photon absorption spectrum because of the different quantum mechanical selection rules governing the light absorption (Xu *et al*., 1996; Bestvater *et al*., 2002; Makarov *et al*., 2008; Drobizhev *et al*., 2011), so in order to optimize the excitation efficiency for fluorescence imaging there is a need to measure the wavelength-dependent excitation efficiency of the dyes. There exist few, and only solution-based, data of wavelength-dependent two-photon excitation efficiency for excitation wavelengths shorter than 680 nm (Bestvater *et al*., 2002; Makarov *et al*., 2008; Drobizhev *et al*., 2011; Li *et al*., 2011). The absorption and excitation spectrum for a dye in solution can be quite different from those when the dye is in a biological environment (Lakowicz, 2006). In particular for nuclear stains such as SYTOX Green and DAPI, which are almost nonfluorescent when not bound to DNA (Kapuscinski, 1995; Roth *et al*., 1997), the solution should at least contain DNA as well as the dye under study. For DAPI, there is also a shift in the absorption spectrum when bound to DNA (Kapuscinski, 1995).

In this paper, we show the wavelength-dependent two-photon excitation efficiency for a number of UV excitable fluorescent dyes, and compare the photo-bleaching rate for the most efficient visible light excitation and excitation within the Ti:Sapphire range. To overcome the limitations of solution-based measurements discussed above, all the data were obtained with the dyes used as fluorescent labels within biological specimens. Additionally, we discuss the intensity-dependent photo-bleaching rate for the commonly used fluorescent marker DAPI. We show that with excitation in the visible wavelength range, the fluorescence emission intensity is up to 10 times higher than that obtained with the most efficient excitation available using the standard near-infrared excitation wavelengths from the Ti:Sapphire laser.

## Experimental

### Sample preparation

We investigated the two-photon excitation spectrum, photo-bleaching behaviour and excitation power dependence of the fluorescence signal for the dyes DAPI, Hoechst 33258, SYTOX Green, AlexaFluor 350 and Calcofluor White.

For investigating DAPI and Hoechst 33258, 3T3-L1 cells were grown at 37°C, 5% CO2 in T75 culture flasks using Dulbecco's modified Eagle's medium (Life Technologies, Carlsbad, CA, USA) supplemented with 10% Foetal Bovine Serum heat inactivated (Life Technologies), 100 units mL^-1^ penicillin and 100 μg mL^-1^ streptomycin (Life Technologies). Cells were regularly passaged to maintain exponential growth. Twenty-four hours before staining, at 80% confluency, cells were treated with StemPro Accutase Cell Dissociation Reagent (Life Technologies), diluted 1:10 with fresh medium (approximately 1 × 10^5^ cells mL^-1^) and transferred to uncoated glass cover slips in 12 well plates (1 μL per well). The 3t3-L1 cells were fixed for 20 min using 3.7% Paraformaldehyde (Sigma-Aldrich, St Louis, MO, USA) in Phosphate Buffered Saline (PBS) pH7.0 (Life Technologies), washed twice in PBS and stored at 4°C in PBS until staining. For staining the cells with DAPI, the cover slips were mounted on slides using Vectashield Hard-Set Mounting Medium with DAPI, 1.5 μg mL^-1^ (Vector Laboratories, Burlingame, CA, USA). For staining the cells with Hoechst, Hoechst 33258 solution (Sigma-Aldrich) was diluted to 2 μg mL^-1^ using PBS and left to stain in the dark at room temperature for 40 min. The cover slip was then mounted using Vectashield Hard-Set Mounting Medium (Vector Laboratories).

The yeast *Saccharomyces cerevisiae* was maintained on YPD agar plates containing 1% Yeast Extract (Oxoid), 2% Peptone (Oxoid, Basingstoke, UK), 2% Glucose (Sigma-Aldrich) and 2% Agar (Sigma-Aldrich), and was incubated at 20°C for 48 h prior to staining. The yeast cells were transferred to a glass cover slip via a sterile swab and stained with Calcofluor White Stain (Fluka Analytical, Buchs, Germany, Sigma-Aldrich) which was prepared and used as described in Sigma-Aldrich data sheet 18909.

All samples were imaged immediately, except the DAPI-stained 3T3 cells which were left to set to avoid specimen movement during measurements of photo-bleaching.

A 16 μm thick cryostat section of mouse intestine stained with Alexa Fluor 350 wheat germ agglutinin, Alexa Fluor 568 phalloidin and SYTOX Green nucleic acid stain (Life Technologies, Fluocell slide #4) was used to investigate the wavelength dependence of the excitation efficiency of AlexaFluor 350 and SYTOX Green.

We confirmed that the staining protocols worked by imaging stained samples on a standard wide-field epifluorescence microscope.

### Two-photon microscopy

**Figure 1:**
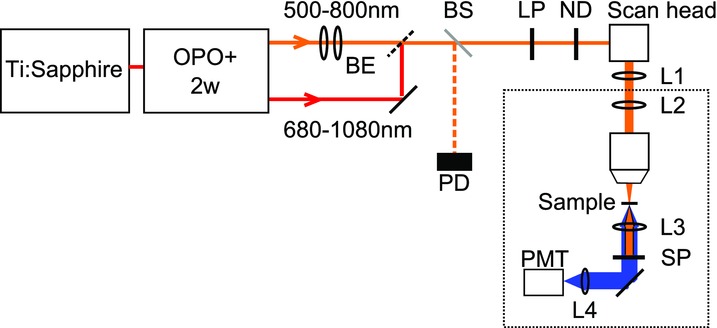
Schematic of the setup for two-photon microscopy. The frequency-doubled OPO signal (500–800 nm) and the residual pump beam (680–1080 nm) were filtered by a long-pass filter (LP). BE, beam expander; BS, beam splitter; PD, photodetector monitoring the laser power; ND, neutral density filter; L1 and L2, scan lens and tube lens. These were different from those used in Norris *et al*. (2012a) to suit the wavelength range used here. L3, condenser lens; SP, filters to remove the excitation light; L4, focusing lens for detector (PMT).

The two-photon excitation experiments were performed using a home-built scanning two-photon fluorescence microscope, based around an upright microscope base (Nikon Eclipse E600FN, Nikon, UK). A full description of this microscope is given elsewhere (Norris *et al*., 2012a) so only a brief description of the microscope and modifications made for this experiment is reported here. A schematic of the setup is shown in Figure 1.

The excitation source was a wavelength-tunable OPO and frequency mixing system (Coherent Chameleon Compact OPOVis, Coherent, Glasgow, UK and APE, Berlin, Germany), pumped by a Ti:Sapphire laser with a repetition rate of 80 MHz. The continuous tuning range of 500–1080 nm that was available by using both the residual pump beam (680–1080 nm) and the frequency-doubled OPO signal (500–800 nm), allowed a comparison between visible and near-infrared excitation. The pulse length was measured using an autocorrelator (Pulsecheck, APE, Fremont, CA, USA) to be 200 ± 20 fs over the entire wavelength range. The frequency-doubled OPO signal beam was shaped using a beam expander. The laser output was filtered with a 515 nm long-pass filter (Schott glass OG515) and the power was adjusted using a neutral density filter.

The excitation light was focused using a 20x/0.75 N.A. dry microscope objective (Nikon, Plan Apo, Nikon) for acquiring the images used to construct the excitation spectra, and a 60x/1.3 N.A. oil immersion objective (Nikon, Plan Apo, Nikon) for acquiring the images used to quantify the photo-bleaching and measuring the excitation power dependence of the fluorescence signal intensity. For imaging the Calcofluor White-stained yeast cells, all data were acquired using the 20× objective. The wavelength-dependent transmission (ca 50%) of the excitation path of the microscope was measured and the data were corrected for this. The average excitation powers (P_exc_) were measured to be 1–20 mW at the specimen plane.

The fluorescence signal was collected in transmission by the condenser and detected using non-de-scanned detection and a photomultiplier tube optimized for short wavelength detection (PMM01, Thorlabs, Ely, UK). The scan speed was 70 lines s^-1^, giving pixel dwell times of 10–20 μs. For all fluorescent dyes used here except SYTOX Green, the fluorescence signal was filtered using a 450/80 nm band-pass filter (HQ 450/80, Chroma Technology, Bellow Falls, VT, USA) and a 533 nm short-pass filter (FF01/355/SP, Semrock, Inc., Rochester, NY, USA). At the shortest excitation wavelengths, the 450/80 nm filter was replaced by a 430/50 nm filter, (HQ 430/50-P, Chroma Technology) to reduce bleed-through of the excitation light. The spectra generated from the images were corrected for the different filter transmissions. We measured these transmissions for each dye by acquiring and comparing images with the different filters at the same excitation wavelength. For SYTOX Green, a 520/15 nm band-pass filter (FF01-520/15–25, Semrock Inc.) was used together with the 533 nm short-pass filter to suit the longer emission wavelength of that dye.

The fluorescence intensity decays used to quantify photo-bleaching were measured by irradiating the specimen continuously and acquiring images from a single focal plane of each specimen over a period of time. We chose to compare the fluorescence intensity decay for the excitation wavelengths that gave the strongest signal within and outside the Ti:Sapphire wavelength range, respectively. The laser power was monitored using a photodetector (DET100A, Thorlabs) and the intensity decays were corrected for the fluctuations in the laser power (a few per cent), averaged over a full frame. A polynomial was fitted to the laser power measurement and then used to correct the fluorescence intensity decays. This corrects for overall changes in laser power as necessary for quantifying the photo-bleaching, but not for faster changes in excitation power within or in between frames. To minimize the impact of photo-bleaching on the excitation spectra, particularly for the shorter excitation wavelengths, each sample area was imaged with only a few wavelengths, in overlapping ranges, instead of imaging a single area at all wavelengths. The data were then normalized between sets. The excitation power was the same for all points in the spectra to within 10%, and we corrected the spectra for these small power differences. For DAPI and Calcofluor White, the measurement points at the shortest excitation wavelengths were measured at half the power to avoid rapid photo-bleaching and the data were corrected for this. As both excitation powers were below the saturation effects visible in the excitation power-dependent measurement, this did not skew the spectral data.

## Results and discussion

**Figure 2:**
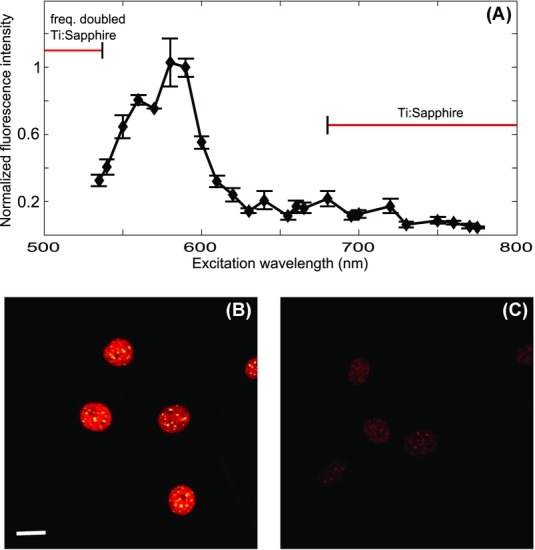
(A) The measured two-photon excitation spectrum for DAPI. The data points are indicated with diamonds and the lines are a guide to the eye. The error bars indicate the SD over the data set. The wavelength range available with the Ti:Sapphire laser alone, and by frequency doubling is indicated in the figure. (B, C) DAPI-stained 3T3 cells imaged with excitation wavelengths of (B) 590 nm and (C) 680 nm at the same excitation power (P_exc_ = 5 mW), for two different sample areas within the same specimen. Scalebar is 20 μm.

The complete excitation spectrum for DAPI is shown in Figure 2(A), clearly showing the more efficient excitation at the visible wavelengths obtained from the OPO, as compared to the wavelength range available with the Ti:Sapphire laser alone (also indicated in the figure). Each data point is averaged over at least 16 cells from 1–2 regions of interest, over 2 different samples. The error bars indicate the SD over the data set. Since the photo-bleaching and sample damage was excessive at the shortest wavelengths available from the laser system, we only studied the excitation wavelength range above 530 nm. Indeed, the measured fluorescence signal intensity was five times higher with an excitation wavelength of 580 nm compared to the shortest available wavelength from the Ti:Sapphire laser, 680 nm, which was also the most efficient excitation wavelength within the range of the Ti:Sapphire fundamental. Fluorescence images of DAPI-labelled nuclei within 3T3 cells excited at: (1) 590 and (2) 680 nm with the same time-averaged excitation power at the specimen plane are shown in Figures 2(B) and (C). Samples that were not stained showed no autofluorescence, and thus we attribute the signal to fluorescence from DAPI.

**Figure 3:**
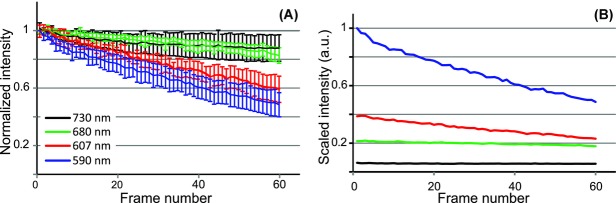
(A) Normalized intensity decays for DAPI-labelled cell nuclei in 3T3 cells excited at four different wavelengths. The wavelength and excitation powers are 730 nm and P_exc_ = 9 mW (black line), 680 nm and P_exc_ = 9 mW (green line), 607 nm and P_exc_ = 10.5 mW (red line) and 590 nm and P_exc_ = 10.5 mW (blue line). The pixel dwell time was 14 μs and the frame rate 0.14 frames/s. The error bars indicate the SD over the data set. (B) The same fluorescence intensity decays plotted as actual signal (not normalized), illustrating that the emission intensity remained higher for a large number of frames for the shorter excitation wavelengths.

As shown in Figure 3(A), the photo-bleaching rate was higher for the shorter excitation wavelengths. Despite this, because of the higher excitation efficiency at the shorter wavelengths, the fluorescence intensity remains higher in the images acquired at those wavelengths for a large number of frames. This is illustrated in Figure 3(B), where the fluorescence intensity decays in Figure 3(A) were rescaled by multiplying with the relative excitation efficiency for that wavelength, in effect, plotted as a (scaled) actual intensity, rather than normalized intensity. The noise in the signal was primarily due to noise in the laser intensity, which was amplified by the intensity-squared dependence of the fluorescence intensity on the excitation power. We only corrected for the power averaged over a frame and any fluctuations within the acquisition time still remained in the data. The error bars in Figure 3(A) represent the SD in the normalized signal for a minimum of 12 cells from 4 areas within 2 samples. The error bars reflect a combination of noise due to intensity fluctuations of the laser and a distribution of photo-bleaching rates between cells.

**Figure 4:**
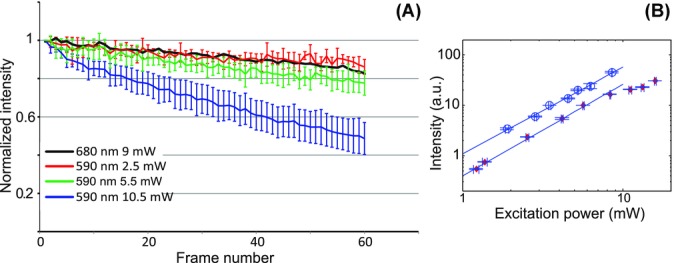
(A) Fluorescence intensity decays for DAPI for 590 nm excitation at powers of P_exc_ = 2.5 (red line), 5.5 (green line) and 10.5 mW (blue line) and for comparison, the fluorescence decay for excitation at 680 nm and P_exc_ = 9 mW (black line, error bars omitted for clarity). The pixel dwell time was 14 μs and the frame rate 0.14 frames/s. (B) Excitation power dependence of the fluorescence signal for excitation at 730 nm (diamonds) and 590 nm (triangles). The solid lines are a fitted linear dependence. The data are from each one sample area containing 2 and 4 cells, respectively. The horizontal error bars represent the uncertainty (<0.25 mW) in the laser power measurement, and the vertical error bars the SD in the normalized fluorescence intensity from the cells in that region of interest.

The photo-bleaching rate also depended on the excitation intensity. Figure 4(A) shows the power-dependent fluorescence signal intensity decays for excitation at 590 nm using excitation powers of P_exc_ = 10.5, 5.5 and 2.5 mW, respectively. The error bars represent the SD in the normalized signal for a minimum of 13 cells from 3 areas within 2 samples. This clearly shows the reduced photo-bleaching rate at lower excitation power, and indicates that the photo-bleaching rate scaled nonlinearly with the excitation power. For comparison, the fluorescence intensity decay for excitation at 680 nm is also shown. For excitation at 680 nm, a lower excitation intensity did not substantially reduce the already low photo-bleaching rate within the measurement uncertainty (data not shown), and increasing the excitation power to increase the signal gave rise to rapid sample damage, which may arise from sample heating.

From our data, we can conclude that for these specimens the photo-bleaching rate scales faster than linear with the excitation power, but not with an order (*n*) higher than 2. This is in contrast to the literature finding orders of *n* > 3 for several fluorophores (Patterson & Piston, 2000; Kalies *et al*., 2011) and *n* = 2 for Hoechst 33342 (Kalies *et al*., 2011). The fact that the photo-bleaching rate is wavelength-dependent and increases at shorter excitation wavelengths is, however, in agreement with Patterson & Piston (2000) and Bush *et al*. (2007). Also, in agreement with Patterson & Piston (2000) and Bush *et al*. (2007), we observe that the photo-bleaching rate is not simply increased for a less efficient excitation wavelength, where the number of excess photons that can lead to further absorption from the excited state is large, nor does it scale with the number of excitation events, that is, the photo-bleaching rates in Figure 3(A) are not proportional to the excitation efficiency in Figure 2(A). Additionally, the photo-bleaching rate can depend on the repetition rate of the laser source (Donnert *et al*., 2007).

Figure 4(B) shows a log-log plot of the excitation power dependence of the fluorescence intensity. A straight line fit to the data (for excitation powers less than P_exc_ = 7 mW) gave a slope of 1.79 ± 0.01 for excitation at 730 nm and 1.72 ± 0.01 for excitation at 590 nm, confirming the two-photon excitation process for both wavelengths. The slope is an average over data from a minimum of 12 cells from 2 areas within 2 samples. At short wavelengths, there was some saturation of the fluorescence intensity at higher excitation powers, evident from the gradually decreasing slope with increasing excitation power. This was not mainly an effect of photo-bleaching, which was verified by acquiring an image at lower excitation power after the data sets were acquired.

**Figure 5:**
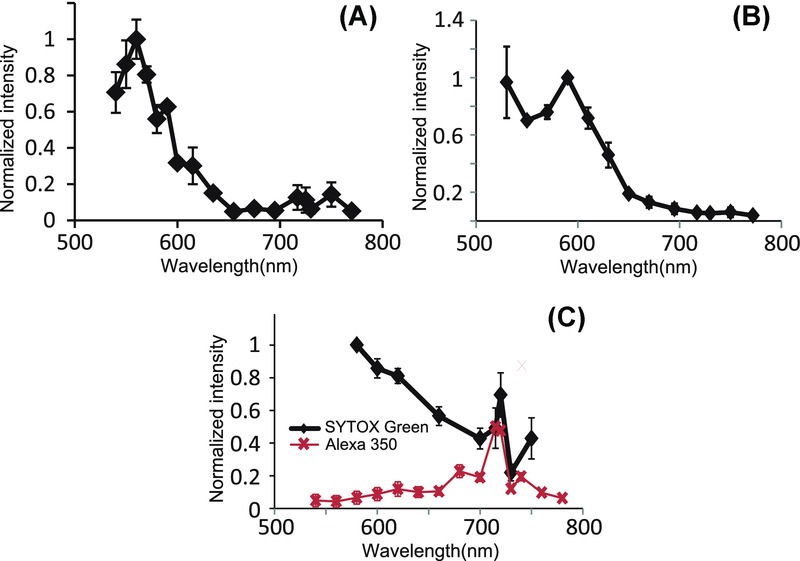
Excitation spectra for (A) Hoechst 33258 with P_exc_ = 5 mW, (B) Calcofluor White with P_exc_ = 2.5 mW and (C) SYTOX Green (black diamonds and line) and AlexaFluor 350 (red crosses and line) with P_exc_ = 10 mW.

The measured two-photon excitation spectra for Hoechst 33258, Calcofluor White and SYTOX Green are shown in Figures 5(A)–(C), respectively. Clearly, also for these fluorescent dyes, the excitation was more efficient for visible wavelengths than for wavelengths above 680 nm. The excitation is up to 7 times more efficient for Hoechst 33258 and up to 10 times more efficient for Calcofluor White for excitation at visible wavelengths as compared to wavelengths longer than 680 nm. However, as can be seen from Figure 5(C), this was not the case for AlexaFluor 350, which was most efficiently excited at around 715 nm. The peak at about 715 nm in the excitation spectrum for SYTOX Green was likely cross-talk from emission from AlexaFluor 350. For Calcofluor White, the drop in excitation efficiency at wavelengths shorter than 590 nm was not expected from its one-photon absorption (Hoch *et al*., 2005), and was possibly partly due to the presence of the counterstain Evans blue in the dye mixture, since that has a one-photon absorption at these shorter wavelengths (Hed & Dahlgren, 1983). This spectrum was acquired at a lower power of P_exc_ = 2.5 mW compared to the other fluorescent dyes, since the photo-bleaching was rapid and there was a clear saturation of the fluorescence intensity for higher excitation powers (data not shown). Each data point in the spectrum is an average over a minimum of 13 cells from 3 areas over 2 samples for Hoechst 33258, hundreds of cells from 2 samples for Calcofluor White, 23 cells from 2 sample areas for Alexa 350, and 100–200 cells from 6 areas for SYTOX Green.

**Figure 6:**
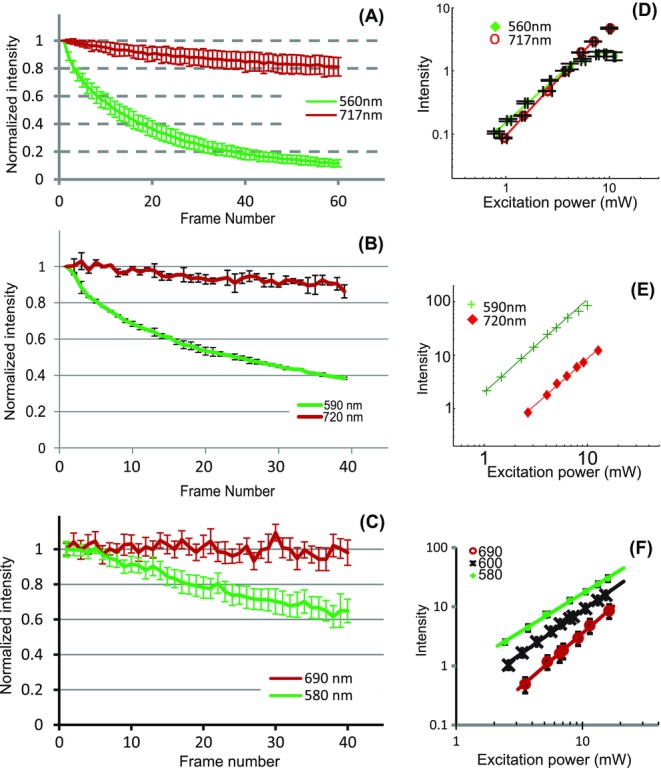
(A) Fluorescence intensity decay for Hoechst 33258 with excitation at 560 nm and P_exc_ = 5 mW (green line) and 717 nm and P_exc_ = 4 mW (red line). The pixel dwell time was 18 μs and the frame rate 0.18 frames/s. (B) Fluorescence intensity decay for Calcofluor White for excitation at 590 and 720 nm, both with P_exc_ = 6 mW. The pixel dwell time was 9 μs and the frame rate 0.09 frames/s. (C) Fluorescence intensity decays for SYTOX Green for excitation at 580 nm and P_exc_ = 10.5 mW and 690 nm, P_exc_ = 10 mW. The pixel dwell time was 18 μs and the frame rate 0.18 frames/s. (D) Excitation power dependence of the fluorescence signal for Hoechst 33258 with excitation at 560 nm (green diamonds) and 717 nm (red rings). The solid lines are a fitted linear dependence. The data are from each one sample area containing 4 and 6 cells, respectively. (E) Excitation power dependence of the fluorescence signal for Calcofluor White for excitation at 590 nm (green crosses) and 720 nm (red diamonds).The solid lines are a fitted linear dependence. Each data set is from one sample area containing hundreds of cells. (F) Excitation power dependence of the fluorescence signal for SYTOX Green for excitation at 580 nm (green crosses), 600 nm (black crosses) and 690 nm (red circles). The solid lines are a fitted linear dependence. The data are from each one sample area containing 10, 12 and 10 cells, respectively.

The fluorescence intensity decays at two different excitation wavelengths for Hoechst 33258, Calcofluor White and SYTOX Green are shown in Figures 6(A)–(C), respectively. For all these fluorescent dyes, as was the case for DAPI, the photo-bleaching is faster for shorter excitation wavelengths. Also, here, despite the higher photo-bleaching rate, a large number of frames can be acquired with stronger signal at the shorter excitation wavelength. The data in Figures 6(A)–(C) are an average over at least 12 cells from 4 areas on 1 sample for Hoechst 33258, hundreds of cells from 2 areas on 1 sample for Calcofluor White, and at least 19 cells from 2 areas for SYTOX Green for each excitation wavelength.

For Hoechst 33258, the photo-bleaching rates were clearly higher than for DAPI, possibly making it a less reliable alternative to DAPI for fixed cells. For SYTOX Green, in order to only compare the fluorescence intensity decay due to emission from SYTOX Green, and avoiding to excite AlexaFluor 350, we chose to acquire the fluorescence decays and study the exc-itation power dependence of the fluorescence signal with excitation at 580 and 690 nm, rather than comparing the excitation wavelengths that gave maximum emission intensity in the visible wavelength range and within the range of the Ti:Sapphire laser. For this sample, the tissue is mounted in an antifade agent by the manufacturer, which reduces the absolute rate of photo-bleaching. Thus, the absolute rate of photo-bleaching cannot be compared with the other dyes. The effect of the antifade agent on the photo-bleaching dynamics is also not clear, and it could be the cause of the more linear intensity decay for short wavelength excitation, as compared to the clearly exponential decay observed for the other dyes studied here. Nevertheless, we still see a difference in the rate of photo-bleaching for different excitation wavelengths.

Log-log plots of the excitation power dependence of the fluorescence signal at two excitation wavelengths for Hoechst 33258, Calcofluor White and SYTOX Green are shown in Figures 6(D)–(F), respectively. The excitation wavelengths are the same as those used for the fluorescence intensity decays. For all the fluorescent dyes, the slope of a linear fit to the data revealed the nonlinear excitation process. For SYTOX Green, however, the slope of the fit was only 1.36 ± 0.03 for excitation at 580 nm, possibly due to substantial competing one-photon absorption. This is corroborated by that at a slightly higher excitation wavelength of 600 nm, which should have substantially smaller one-photon absorption cross-section, the slope of the fitted line was 1.53 ± 0.04, indicating a more nonlinear absorption process. Note that we are, nevertheless, detecting the emission resulting from the two-photon excitation process at 580 nm, since the detection wavelength is clearly shorter than the excitation wavelength. The photo-bleaching rate for excitation at 580 nm was similar to that at excitation with 600 nm (data not shown), indicating that the photo-bleaching rate was not substantially affected by the one-photon absorption at 580 nm. The slope of the fitted line for excitation at 680 nm was 1.76 ± 0.01. For AlexaFluor 350 and excitation at 730 nm, the slope of the fitted line was 1.98 ± 0.01 (data not shown). For Calcofluor White, the linear fits gave gradients of 1.8 ± 0.2 and 1.89 ± 0.04 for excitation at 590 and 720 nm, respectively, and for Hoechst 33258 the gradient of the straight line fit for excitation powers less than P_exc_ = 5 mW was 1.56 ± 0.01 and 1.7 ± 0.01 for excitation at 560 and 717 nm, respectively. For Hoechst 33258, substantial saturation effects were visible also at moderate excitation powers for excitation at the shorter wavelength. By contrast to the fluorescence intensity saturation visible for DAPI, here the apparent saturation is partly due to photo-bleaching.

The slopes are averaged over 11 cells from 3 areas over 2 sample for excitation at 560 nm and 12 cells from 2 areas on 1 sample for excitation at 717 nm for Hoechst 33258, over hundreds of cells from 2 areas on 1 sample for each of the excitation wavelengths for Calcofluor, and over 20 cells from 2 areas excitation at 580 and 690 nm and over 12 cells from 1 area for excitation at 600 nm for SYTOX Green. In the log-log plots, the horizontal error bars represent the uncertainty in the laser power measurement, and the vertical error bars the SD in the normalized fluorescence intensity from the cells in that region of interest.

Finally, we point out that although the scattering and one-photon absorption in tissue would be somewhat higher at these shorter wavelengths, as compared to the wavelength range of about 700–1000 nm usually employed in two-photon microscopy, this is not necessarily a large effect, since in biological materials the relevant scattering is Mie scattering rather than Rayleigh scattering, and so is not as strongly dependent on wavelength. Additionally, these effects are likely offset by the more efficient excitation of the dyes at these wavelengths. Nevertheless, employing short-wavelength two-photon excitation for these UV excitable chromophores allows efficient excitation with wavelengths much more benign than UV light.

## Conclusion

In conclusion, we have shown that for a number of common UV excitable fluorescent dyes; the nuclear stains DAPI, Hoechst 33258 and SYTOX Green and chitin- and cellulose-staining dye Calcofluor White, there is an increased excitation efficiency (of up to 10-fold) when visible wavelengths are used for two-photon excitation, as compared with using the wavelength range available directly from a Ti:Sapphire laser (680–1080 nm). We also find that although the rate of photo-bleaching increases at shorter wavelengths, it is still possible to acquire many images with a higher signal level, making this excitation regime potentially interesting for rapid images of samples, and allowing use of lower excitation intensities, which could be beneficial for imaging live specimens.
